# Evaluation of various traditional machine learning techniques for predicting the acute effect of different hamstring muscle stretching methods among male soccer players

**DOI:** 10.1038/s41598-025-27338-6

**Published:** 2025-12-04

**Authors:** Elham Hosseini, Mohammad Alimoradi, Mojtaba Iranmanesh, Sahar Zaidi, Arian Azizian, Andreas Konrad, Hadis Mohseni

**Affiliations:** 1 Department of Sports Injuries and Corrective Exercises, Faculty of Sports Sciences, Shahid Bahonar University of Kerman, Kerman, Iran; 2 HERC – Health, Exercise & Research Center, Mina Rashid, Dubai Maritime City, Dubai, United Arab Emirates; 3 Department of Physiotherapy, School of Nursing Sciences and Allied Health, Jamia Hamdard, New Delhi, India; 4 Department of Computer Engineering, Shahid Bahonar University of Kerman, Kerman, Iran; 5 Institute of Human Movement Science, Sport and Health, Graz University, Graz, A- 8010 Austria

**Keywords:** Supervised machine learning, Athletic performance, Stretching exercises, Hamstring muscles, Computational biology and bioinformatics, Engineering, Health care, Mathematics and computing

## Abstract

This study investigated the acute effects of static (SS), dynamic (DS), and ballistic (BS) hamstring stretching on performance in male soccer players and applied machine learning (ML) to predict protocol efficacy. A total of 249 players with and without hamstring shortening completed each protocol across three sessions with 72 h of rest. Hamstring shortening classified via passive knee extension test (> 32.2° knee angle). Flexibility, strength, sprint, power, and agility were measured pre- and post-stretching. Each protocol: 4 sets × 30 s (holds/swings/bounces at 50–60 bpm), 10 s rest. ML models (k-NN, SVM, random forest) were trained on pre–post difference scores, with feature selection applied to identify key predictors and Synthetic Minority Over-sampling Technique used to address class imbalance. Findings indicate SS optimally acutely improves flexibility, whereas DS offers broader immediate performance benefits for a subsequent activity. Combining feature selection and data balancing increased k-NN accuracy to 53% (only ~ 20% points above the chance level of 33.3% for this three-class problem), highlighting methodological challenges in predicting individual responses. Exploratory analysis using ML using synthetic minority over-sampling technique reached a peak accuracy of 53.06% (compared to a baseline of 33.3%), demonstrating the promise of the approach but also highlighting the challenges of applying ML to predict individual responses to stretching interventions, underscoring the need for larger datasets and more advanced models.

**Supplementary Information:**

The online version contains supplementary material available at 10.1038/s41598-025-27338-6.

## Introduction

 The hamstring muscle group is fundamental to athletic performance, particularly in soccer, where success hinges on explosive actions such as sprinting, jumping, and rapid changes of direction^1,2^. For athletes to express their full potential, the hamstrings must not only generate substantial force but also function effectively through a complete range of motion (ROM)^1^. This interdependence of strength and flexibility is crucial; for instance, greater hamstring length permits a more extended stride during a sprint, contributes to the powerful extension of the hip and knee in a jump, and supports the controlled, forceful deceleration needed to change direction efficiently^1,3^. Conversely, restrictions in flexibility—often manifested as muscle tightness—can compromise this intricate biomechanical harmony. Such limitations may diminish movement economy, disrupt technical form, and ultimately curtail performance. Perhaps more critically, insufficient hamstring flexibility is a recognized risk factor for injury^3^. When the muscle-tendon unit lacks the extensibility to accommodate high-velocity elongation—such as during the terminal swing phase of a sprint—it becomes susceptible to strains. Therefore, for soccer athletes and the professionals who support them, nurturing optimal hamstring function extends beyond the pursuit of performance excellence; it is also an essential component of sustaining athlete health and availability throughout the competitive season^3,4^.

Muscle stretching is one of the most commonly used techniques in athletic preparation to acutely improve flexibility and performance^5^. Stretching is often incorporated into warm-up routines with the goal of enhancing joint ROM, promoting muscle activation, and preparing the neuromuscular system for subsequent activity^6^. Several stretching methods are widely practiced, including static stretching (SS), dynamic stretching (DS), and ballistic stretching (BS), each with distinct mechanical and physiological effects^7^. SS involves holding a muscle in a lengthened position for a fixed duration, typically without movement. It is generally considered easy to perform and effective for increasing ROM; however, some studies have suggested that prolonged SS may temporarily reduce muscle strength and power output, which can be detrimental to performance when applied immediately before high-intensity efforts^8,9^. In contrast, DS consists of controlled, rhythmic limb movements through the active ROM, which can enhance flexibility while simultaneously increasing muscle temperature, neural drive, and dynamic motor control. DS has been shown to have positive effects on explosive performance tasks such as sprinting and jumping, making it particularly suitable for use in pre-competition settings^8,10^. BS, a more intense variant of DS, involves bouncing or jerking movements that exceed the usual ROM limits. While this technique may promote increased tendon compliance and elastic energy storage, it also imposes greater mechanical stress on the musculotendinous units. When applied appropriately, BS may provide acute improvements in performance by enhancing the stretch-shortening cycle, but it must be used with caution, particularly in athletes with limited flexibility or neuromuscular control^11–13^.

Although all three stretching modalities are widely used in practice, the literature presents mixed findings regarding their comparative effects on athletic performance. Some studies have found SS to be detrimental to strength and power, while others report minimal or no impact. The disparity in findings across the literature may be attributed to variations in stretching parameters. Specifically, protocols utilizing more moderate intensity and shorter duration appear less likely to compromise subsequent performance^9,14^. DS is generally supported as beneficial for performance, but the degree of improvement can vary depending on the type and duration of stretching^13^. BS is less commonly used in structured programs due to its more aggressive nature, yet it may yield superior effects in tasks requiring explosive strength and power^14^. Moreover, inter-individual differences such as baseline flexibility, training age, and limb dominance can influence the effectiveness of each stretching modality. As a result, a one-size-fits-all approach to stretching is often suboptimal, and more personalized strategies may be required^9,10,14^. In light of these complexities, modern analytical tools such as machine learning (ML) offer promising solutions for modeling the relationships between stretching modalities and performance outcomes^15^. ML techniques can manage large and multidimensional datasets, uncover complex patterns, and generate predictive models based on individual characteristics and test results^16^^17^. Compared to traditional statistical methods, ML algorithms can adaptively learn from data, offering a more nuanced understanding of how different variables interact to affect performance. In particular, ML can be useful in identifying which stretching techniques are likely to be most effective for athletes with specific profiles, ultimately supporting further exploration of training interventions^17,18^.

Given this context, the present study was designed to investigate the acute effects of SS, DS, and BS protocols on a range of performance-related outcomes in male soccer players. Additionally, the study examined how traditional ML algorithms could be used to model and predict the effectiveness of different stretching protocols based on individual performance data.

## Results

### Class distribution within protocols

**Fig. 1:**
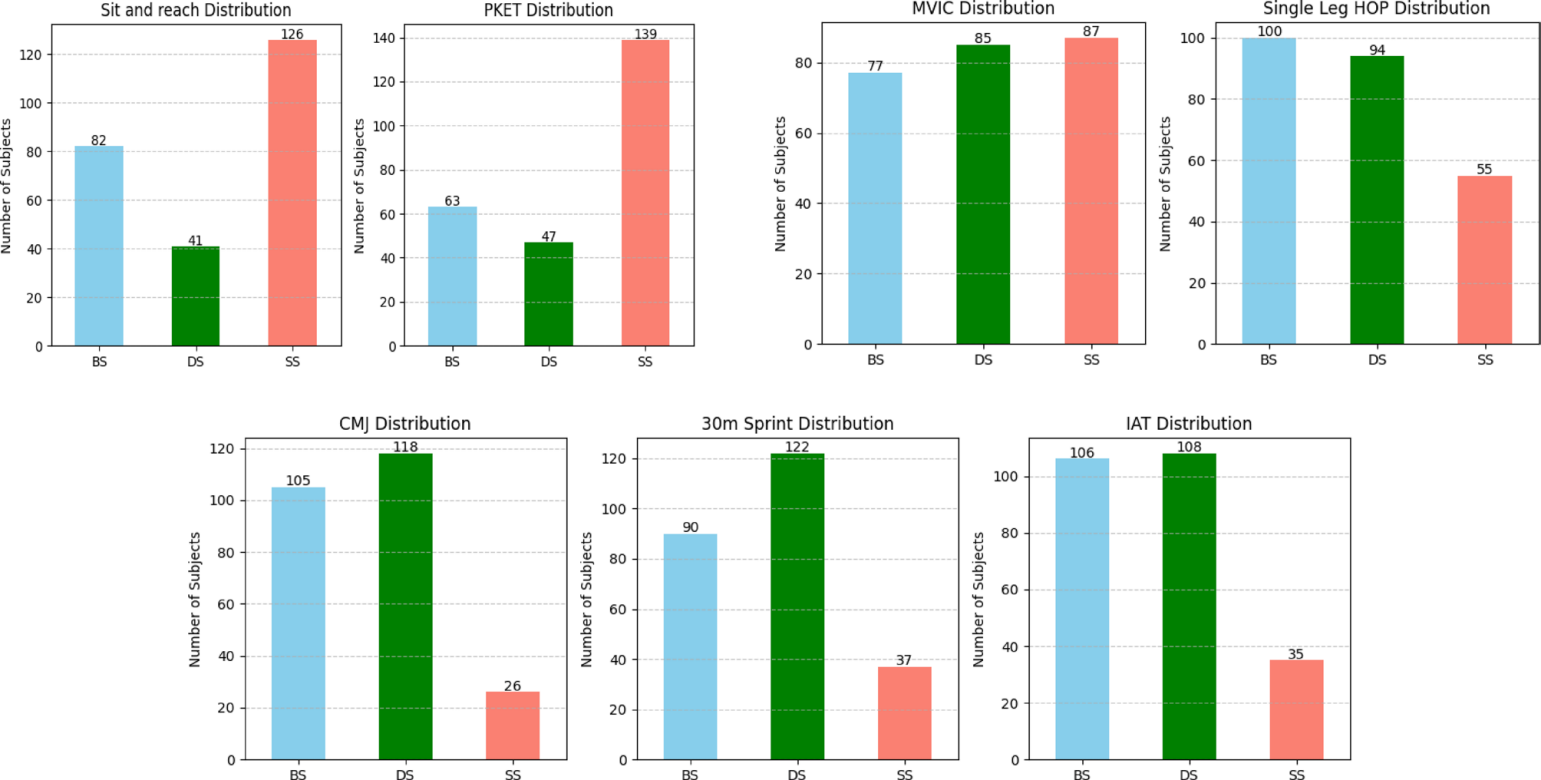
Bar plot of mean pre-post changes. Shows the effectiveness of each protocol per test. Abbreviations: SS, static stretching; DS, dynamic stretching; BS, ballistic stretching; PKET, passive knee extension test; MVIC, maximal voluntary isometric contraction; CMJ, countermovement jump; IAT, Illinois agility test.

 The analysis of outcomes by protocol revealed distinct effects. For flexibility measures, SS yielded the largest improvements, evidenced by the greatest effect sizes in both the SR and PKET. In contrast, for performance tests including the single-leg hop, CMJ, sprint, and the IAT, DS and BS were more effective. Specifically, DS produced the most substantial acute enhancements across these performance metrics (Fig. 1). The magnitude of these acute effects, expressed as Cohen’s *d* with 95% confidence intervals, is presented in Tables S1-S4 .

### Feature selection

**Fig. 2:**
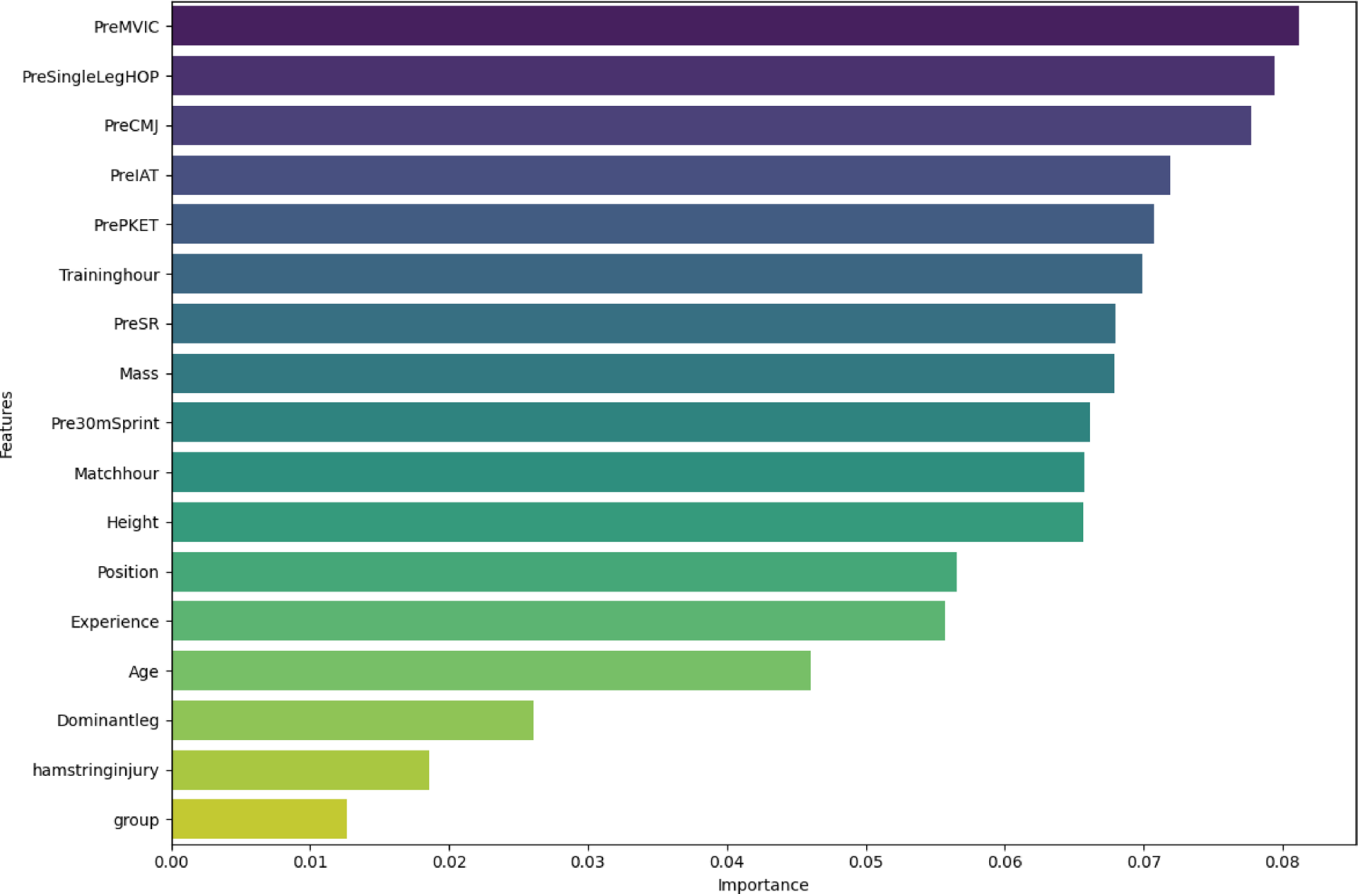
Feature importance ranking -by Extra Trees classifier, determines how each feature contributes in classificationand their level level of importance. Abbreviations: MVIC, maximal voluntary isometric contraction; CMJ, countermovement jump; IAT, Illinois agility test; PKET, passive knee extension test; SR, sit and reach.

As previously mentioned, it is possible that by removing some features and selecting others, models can perform slightly better. By ranking the contribution of most important features in current classification task with Extra Trees in Fig. 2 and knowing the most correlated features from Fig. 6, it can be seen that some features such as ‘group’ with low importance probability and high correlation, may not have the expected positive effect and can decrease the performance of some models, such features better be removed from training variables. The results of classifying with and without feature selection (FS) are shown in Table 1.

### Balanced data

In this study, both balanced and original (imbalanced) data were considered in modeling. As mentioned, data balancing can improve model performance to better by making data equally available for each class during the learning process. the other hand, data augmentation techniques can make model performance better by providing them more variants of data available in learning. The result of modeling with and without balanced data (BD) can be seen in Table 1.

### Evaluation of models

In Table 1 an overall evaluation of the models is different situation shown, as expected models trained on balanced data with selected features generally demonstrated improved performance. For this three-class classification problem, the chance-level accuracy is 33.3%. The highest observed accuracy was 53.06% for the k-NN model with feature selection and balanced data.

**Table 1:** Results of the experiments that were done with and without data balancing and feature selection on various traditional machine learning models.

Model	Accuracy	Precision	Recall	F1-Score
LDA	0.4374	0.4232	0.4374	0.4197
*k*-NN	0.3537	0.3618	0.3537	0.3514
SVM	0.3656	0.3065	0.3656	0.3239
LR	0.4336	0.4232	0.4336	0.4205
Random Forest	0.3894	0.3527	0.3894	0.3623
Extra Trees	0.3574	0.3342	0.3574	0.3408
LDA + FS	0.4654	0.4472	0.4654	0.4465
*k*-NN + FS	0.3534	0.3681	0.3534	0.3528
SVM + FS	0.3571	0.3032	0.3571	0.3160
LR + FS	0.4655	0.4479	0.4655	0.4491
Random Forest + FS	0.3891	0.3624	0.3891	0.3637
Extra Trees + FS	0.3771	0.3580	0.3771	0.3586
LDA + BD	0.4796	0.4873	0.4796	0.4774
*k*-NN + BD	0.4592	0.4466	0.4592	0.4469
SVM + BD	0.4694	0.4740	0.4694	0.4642
LR + BD	0.5000	0.5067	0.5000	0.4979
Random Forest + BD	0.5068	0.5115	0.5068	0.4997
Extra Trees + BD	0.5272	**0.5299**	0.5272	**0.5222**
LDA + FS + BD	0.4524	0.4452	0.4524	0.4417
*k*-NN + FS + BD	**0.5306**	0.5212	**0.5306**	0.5188
SVM + FS + BD	0.4694	0.4763	0.4694	0.4650
LR + FS + BD	0.4354	0.4258	0.4354	0.4245
Random Forest + FS + BD	0.4898	0.5016	0.4898	0.4877
Extra Trees + FS + BD	0.4864	0.4964	0.4864	0.4801

Abbreviations: LDA, linear discriminant analysis; *k*-NN, $\:k$-nearest neighbor algorithm; SVM, Support Vector Machine; LR, logistic regression; BD, balanced (augmented) data; FS, feature selection.

## Discussion

The study’s findings show that different hamstring stretching protocols have different acute effects on various physical performance tests in male soccer players with and without hamstring shortness. SS was associated with greater acute improvements in SR and PKET than BS or DS, while both showed better results in tests like single leg hop, CMJ, sprint, and IAT. As an exploratory proof-of-concept, we applied ML to model the effectiveness of the different acute stretching protocols. Using the Extra Trees classifier, feature selection analysis revealed that several features, such as highly correlated features like ‘group’ showed low predictive value in Extra Trees rankings, supporting the removal of redundant variables to avoid noise. This suggests that eliminating such features enhances classification results. The study additionally investigated at the ways balanced versus imbalanced data affected model training, showing that equal representation during learning through class balancing enhanced model performance. Model evaluation using conventional ML techniques showed that models with feature selection and data balance typically produced higher F1-scores, accuracy, precision, and recall. Particularly, when feature selection and balanced data used together, the k-NN model showed the greatest accuracy (0.5306) and consistent metric improvements. While SVM and Extra Trees benefited less from these preprocessing methods, logistic regression and linear discriminant analysis also demonstrated significant improvements. Overall, the results indicate that when evaluating the impact of hamstring stretching techniques on male soccer players, machine-learning models perform better when features carefully chosen and data is balanced.

This study’s findings provide important updated insights into the relative acute effects of SS, DS and BS on key performance parameters in male soccer players with and without hamstring shortness. According to current high-quality studies, including a comprehensive systematic review and meta-analysis various stretching techniques produce comparable acute improvements in flexibility measures such as the SR and PKET^19,20^. In addition, Systematic evaluations suggest that SS-induced ROM improvements may involve mechanisms such as greater stretch tolerance and decreased passive muscle stiffness^21,22^. While our findings align with the broader pattern of SS enhancing flexibility, the specific contributions of these mechanisms in our cohort remain to be investigated. However, when tested using the IAT, sprint, single hop test, and CMJ, DS and BS procedures showed better effects on power, sprint, agility, and jumping performance. According to current meta-analyses, DS improves neuromuscular activation, muscle temperature, and proprioception, which increases explosive athletic capacities without having the negative acute impacts on strength and power as seen frequently in SS^23^. In other words, this deeper insight highlights the need for a refined approach. DS may be emphasized during pre-competition warm-ups to ensure optimal performance, while SS continues to play an important role in a comprehensive athletic program, promoting long-term flexibility and resilience against injuries^24^. Although BS isn’t as frequently advised because of the possibility of damage, it may significantly increase the efficiency of the stretch-shortening cycle, which could lead to gains in plyometric and sprint outputs^25,26^. As a result, DS prioritized in warm-up routines since it is the most generally advantageous modality for sport-specific performance metrics that call for power and velocity^25^. In the present study, DS demonstrated beneficial acute effects on performance metrics such as sprinting, jumping, and agility, whereas SS was primarily effective for improving flexibility measures. Which is consistent with recent reliable guidelines that discourage long-duration SS before competition in favor of dynamic protocols for optimal acute readiness^20^. This advanced understanding illustrates how various stretching techniques catered to the requirements of the athlete and the sport may be needed for flexibility and explosive performance^11,13,20–23,26,27^.

The ML analysis in this study should be interpreted strictly as an exploratory proof-of-concept. The predictive performance was modest at best (maximum accuracy: 53.06%, only ~ 20% points above the chance level of 33.3% for this three-class problem), yielding limited methodological insights rather than practical predictions. The results of the Extra Trees classifier demonstrated that highly correlated features do not always improve model performance; in fact, if redundant or irrelevant information, such as ‘group,’ remains, they may actually worsen predictive accuracy. This supports recent research in ML that emphasizes careful feature selection to reduce noise and overfitting^28,29^. This study shows that class balancing improves classification performance in multi-class and imbalanced datasets when applying ML methods. Balanced training data prevents bias toward majority classes and helps models learn decision boundaries for all outcomes. This preprocessing procedure improves accuracy, precision, recall, and F1 measures in sports performance modeling^29–31^. K-Nearest Neighbors (k-NN) had the highest accuracy of 53.06% when paired with feature selection and balanced data, indicating its sensitivity to well-curated data and balanced class representation—though still far from practically useful levels. Logistic Regression and Linear Discriminant Analysis also improved, suggesting potential in structured, statistical sports datasets under ideal conditions. While less preprocessed, Support Vector Machines and ensemble tree-based approaches like Random Forest and Extra Trees performed reasonably^17,29,30^. Recent research comparing multiple classifiers for sports prediction tasks suggests that simpler, distance-based models may outperform more complex classifiers when data quality improves^29^. Furthermore, while techniques like SMOTE improved metric scores by addressing class imbalance, it is important to note that it generates synthetic data, while SMOTE balancing improved metrics, it relies on synthetic samples that do not replace real data and can yield an overly optimistic view of performance, as it may not reflect true class distributions. These analyses demonstrate that with improved data preprocessing, ML could potentially aid in personalizing warm-up routines. However, the current accuracy indicates that much more work, including larger and richer datasets, different feature sets (e.g., neuromuscular data), and external validation, is needed to achieve robust predictive accuracy for practitioners^30,31^.

The study highlights the need for evidence-based stretching regimens customized to athletic performance aims, alongside better data preprocessing in ML models to explore stretching outcomes. This exploratory analysis shows that improved data preprocessing modestly boosts performance, but much more work—including larger datasets and advanced models—is needed to achieve any robust predictive accuracy. However, this study has numerous key limitations that should be addressed when evaluating the results. Nevertheless, the stretching discussions were acute, which may limit the potential to capture long-term physiological alterations in flexibility and performance assessments. Research suggests that continuous stretching programs lasting several weeks needed to cause major morphological changes in muscle-tendon units and sustained ROM improvements^32^. Thus, acute stretching durations may have limited flexibility gains in amplitude and durability. Moreover, grouping individuals by ROM limitation in PKET is also challenging. Categories can oversimplify the complicated continuum of flexibility limitations and obscure inter-individual variability within groups, reducing analysis granularity and sensitivity. This technique may also cause variation within groups and affect result comparability, especially when muscle function and daily activity differ substantially across people with similar ROM categories. These limitations suggest that future research should use longer intervention periods and more detailed participant stratification to better understand how stretching methods affect flexibility and functional performance. Several limitations of this study should be considered. First, the findings relate only to the acute effects of stretching; long-term adaptations in muscle architecture and performance require chronic intervention studies. Second, the classification of ‘hamstring shortness’ was based on a single cut-off value from the PKET, which, while practical, may oversimplify a complex continuum of flexibility and obscure individual variations within groups. Finally, the sample consisted exclusively of adolescent male soccer players, which limits the generalizability of the findings to other populations, including females, older athletes, or those in different sports^32,33^.

In conclusion, this study aimed to compare the acute effects of SS, DS, and BS protocols on flexibility and performance outcomes in male soccer players with and without hamstring shortness. The findings suggest that SS is most effective for improving flexibility measures, while DS resulted in better acute performance in metrics such as sprint, agility, and jumping ability. The study highlights that evidence-based stretching regimens should be customized to athletic performance goals. Furthermore, this exploratory proof-of-concept demonstrates that rigorous data preprocessing modestly improves ML performance, but the modest accuracy (53.06%, only ~ 20% points above chance) indicates that more robust models, larger datasets, and different feature sets are needed before any reliable predictions for practitioners could be feasible. Additionally it is important to note that while SMOTE mitigates class imbalance, it generates synthetic data, which may not fully capture the underlying complex distributions of the original minority class, potentially introducing noise. It’s important to highlight that researchers view this aspect as a limitation of SMOTE.

## Methods

### Study design and participants

The Jamia Hamdard Institutional Ethics Committee (11/24 (10/10/2024)) gave its approval to the ethics principles in current research. The authors adhered to the Declaration of Helsinki’s guiding principles. This cross-sectional study designed to assess acute effects, which included a sample of 574 soccer players from club sports in Iran, carried out on September of 2024. Initially, 574 male soccer players under the age of 18 participated in the assessments. From this group, 249 (age = 16.2 ± 0.6 years; height = 168.7 ± 8.2 cm; mass = 59.7 ± 5.8 kg) were selected as subjects for the current study. Participants and their parents informed about any potential dangers associated with the current procedures, and then completed a written informed consent. All subjects divided into two groups: soccer players with hamstring shortening (*n* = 123) and without hamstring shortening (*n* = 126) randomly performed SS, DS, and BS exercises on three consecutive days at an interval of 72 h between each session. All participants had been training regularly, at least three times a week, for a minimum of three years. Inclusion criteria involved athletes under the age 20 with at least three years of regular training experience in a sport, excluding criteria those with muscular or skeletal injuries. Participants instructed to avoid physically demanding activities or consume stimulating substances 24 h before data collection. They followed a similar lifestyle and observed by researchers during training. Two sports science specialists evaluated the athletes using a blind process related to each participant’s stretching state.

### Procedure

**Fig. 3:**
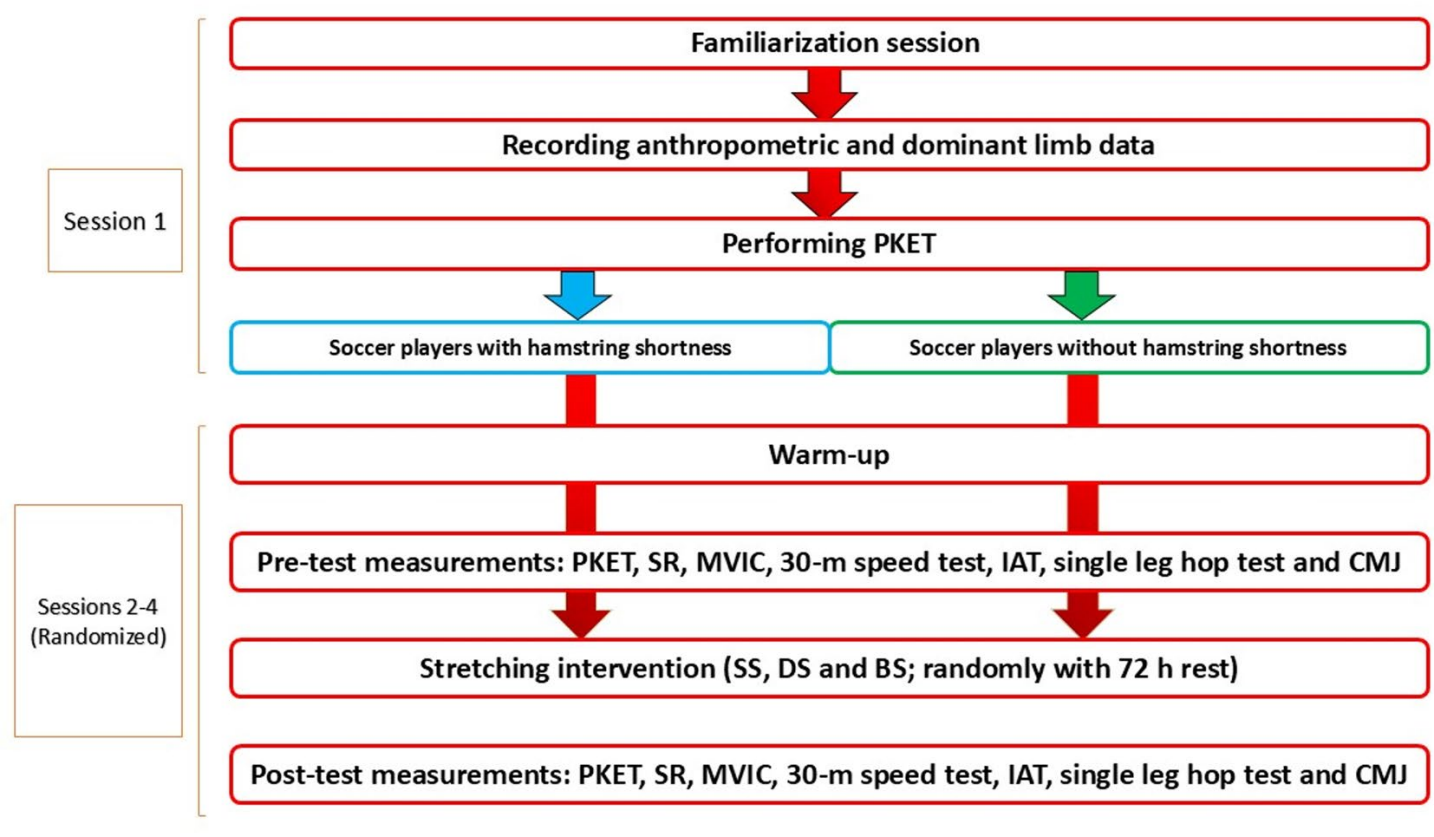
Experimental design showing participant grouping, randomized stretching interventions, and pre- and post-test performance evaluations across three sessions. Abbreviations: PKET, passive knee extension test; SR, sit and reach test; MVIC, maximal voluntary isometric contraction, IAT, Illinois agility test; CMJ, countermovement jump.

After understanding about the test’s procedure during a familiarization session, each participant or legal responsible completed the personal and consent forms 48 h prior to the testing session. Initially on first testing session the anthropometric factors were recorded and then dominant limb was identified by asking participants to kick the ball^31^. After these tests, a passive knee extension test (PKET) performed to identify patients with and without hamstring shortening in their dominant leg. The following were the cut-off values for hamstring shortening: For males, the passive knee extension angle is more than 32.2 degrees^32^. Following divided participants to two groups (with and without hamstring shortness), the subjects were randomized using Rando-web online tools performed three interventions randomly on three consecutive days with 72 h of rest between sessions (SS, DS and BS) served as to prevent carry-over effects between the different stretching modalities. The assessors were blinded to the participants’ subsequent group allocation during this test. In order to investigate the effect of the stretching protocols, all participants performed before and after each stretching intervention session PKET, sit and reach test (SR), maximal voluntary isometric contraction (MVIC), 30-m sprint test, Illinois agility test (IAT), single leg hop test and countermovement jump (CMJ) test as pre-test and post-test evaluations (Fig. 3).

### Stretching protocols

**Fig. 4:**
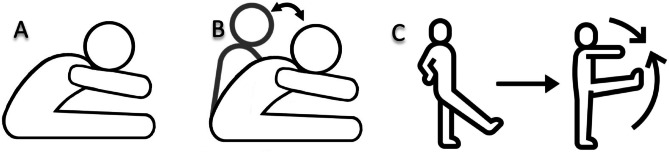
Demonstration of hamstring stretching protocols: **(A)** Static stretching in a seated figure-four position, **(B)** Ballistic stretching with rhythmic bouncing using a metronome, and **(C)** Dynamic leg swings performed at controlled tempo.

Subjects performed the bilateral seated hamstring stretch while sitting with their legs extended. They then leaned forward, flexed their hips, and reached their hands toward their toes until they felt a stretch in their hamstrings. The SS protocol consisted of 4 sets of 30-second holds, with a 10-second rest between sets. For BS, subjects performed the same stretches as previously described for SS. However, instead of holding the stretch, subjects were instructed to get into the specific stretch position until a strong stretch sensation was felt. Within 2 s of feeling a stretch sensation, subjects bounced through the movement at the end of ROM at a rate of one bounce per second for a total of 30 s. To perform the BS, a metronome was set at 60 bpm, and subjects bounced to the beat of the metronome. In addition, the BS protocol involved 4 sets of 30 bouncing movements (one bounce per second, paced by a metronome at 60 bpm) at the end of the ROM, with a 10-second rest between sets. (Fig. 4B)^15,28^. Additionally, the DS for the hamstrings involved raising the leg (from the starting standing posture, one leg is flexed from the hip joint and advanced towards the trunk with the knee completely extended). In DS protocols, the identical exercises performed in the dynamic mode to stretch muscles, whereas in SS and BS regimes, the exercises conducted in postures that reached their maximum ROM. In other word these protocols were performed to the point of mild discomfort but not pain (Fig. 4B, C)^15,35^. The DS protocol involved 4 sets of 30-second periods of continuous, controlled leg swings (approximately 15 repetitions per leg per set) at a tempo of 50 beats per minute, with a 10-second rest between sets (Fig. 4C). Furthermore, every participant took a 5-minute break between the stretching exercise and the post-test evaluations.

### Outcome measures

#### Flexibility and ROM

Hamstring flexibility evaluated using the PKET and SR. The PKET hip flexed at to 90 degrees while the participants were lying supine with the contralateral leg extended on the table. Using a manual goniometer positioned in relation to the lateral epicondyle of the femur, lateral malleolus, and greater trochanter, the examiner evaluated the knee angle after passively extending the knee to the maximum possible stretch while keeping the hip position. This procedure was repeated three times for each limb, and the average of the three measurements was used for statistical analysis. The PKET has a high intra-tester reliability; with interclass correlation coefficients (ICC) reported 0.98^36,37^. The study assessed trunk and lower extremity ROM using the SR test using Baseline^®^. Participants were instructed to sit with their trunk flexed at a 90° angle, rest their heels on the tool, push the instrument with their fingertips without lifting their knees, and extend as far forward as possible. The mean three attempts at the SR measurement were calculated, resulting in a score of 0.98 ICC^38^.

#### MVIC

Hamstring MVIC in the dominant limb recorded using hand-held dynamometer (Lafayette manual muscle testing system model 01163; Lafayette Instrument Company, Lafayette, IN). The dominant leg was tested. The dynamometer was secured to the leg using a standard therapy bed with an upstretched strap, and participants completed three MVIC for 5 s, with a 60-second rest following each trial. The tests were performed for hamstring muscle strength in a prone position, with the strap fastened to limit knee flexion to 85 degrees during extensor and flexor contractions, and the maximum force in kilograms was recorded for each test (ICC = 0.75–0.83)^23,39^.

#### Power and ballistic function

Hamstring power and ballistic function will be measured using single leg hop and CMJ tests before and after the intervention. This study conducted a single-leg hop distance test; thus, participants performed three successful jumps, and then maintaining their landing position for three seconds. The best scores were considered the single-leg hop distance, and arms usage was not restricted during the test (ICC = 0.92)^40^^41^. This study used the CMJ technique, which involves standing upright with hands on hips, to assess jump height. Participants moved their knees rapidly to 90° flexion, followed by a vertical upward movement. The Jump-and-Reach Method with Smart phone and My Jump 3 App was used, which is a quick and cost-effective method for evaluating jump height, as it has a high ICC value of 0.97^42^.

#### Running and agility

Three maximum 30-meter linear sprints were completed by the participants, with a one-minute break in between. Each sprint began 0.5 m ahead of the first timing gate, which was placed at the participants’ hip height. The times were recorded utilizing timing gates system (Smart-speed, Fusion Sport, Australia). The fastest time of the three trials was recorded for the final analysis (ICC = 0.93–0.98)^43^. The IAT was used to evaluate agility and function in a soccer field (ICC = 0.85–0.98)^44,45^. The IAT was conducted on a field measuring 10 m in length and 5 m in width. Four cones were positioned in the center of the field, spaced 3.3 m apart, serving as the start line, finish line, and two turning points. Upon hearing the command “Go,” participants sprinted between the cones as fast as possible. A trial was deemed valid if the participant avoided knocking over any cones and successfully crossed the finish line. The test took place on a football field, and completion times were recorded using timing gate system^1,46^.

## Data preprocessing

### Data scaling and normalization

The purpose of such a task is to scale and standardize the data points before using data for modeling. Scaling data should be within a specific order to ensure that features contribute well and equally to the ML models, especially for those that rely or gradient based optimizations. Scaling features makes them comparable and improves convergence speed, accuracy and model interpretability across different algorithms. In this study z-score method was performed^47^. It centers data around zero with standard deviation of one scaling data in the range of [0, 1].

Formula: $\:z-score=\:\frac{(x\:-\:\mu\:)\:}{\sigma\:}$, where $\:x$ is data points, $\:\mu\:$ is the mean of data and $\:\sigma\:$ is standard deviation.

### Features correlation coefficient

**Fig. 5:**
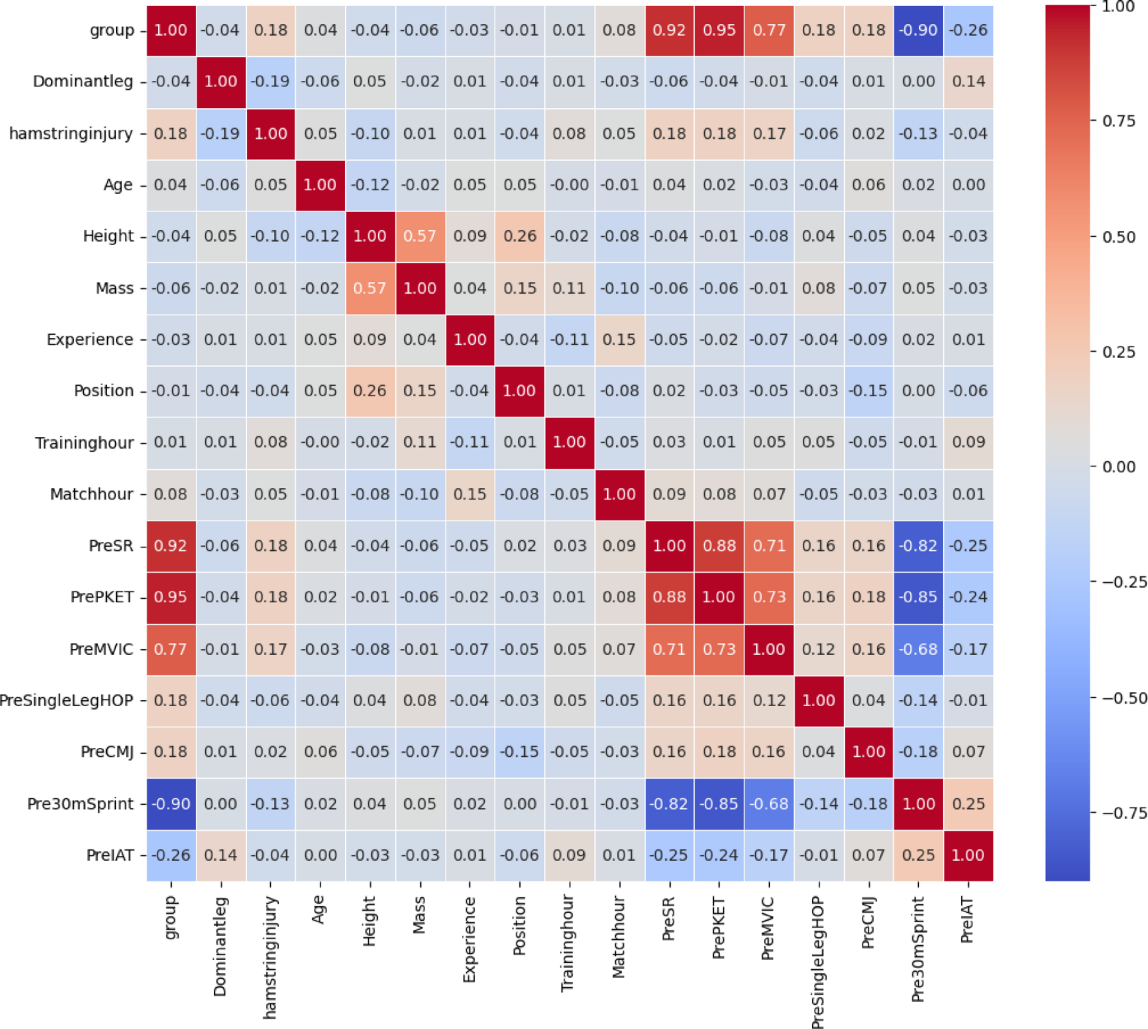
Heatmap of the Pearson correlation coefficient of independent features illustrates the linear relation between each two pair of features. Abbreviations: SR, sit and reach; PKET, passive knee extension test; MVIC, maximal voluntary isometric contraction; CMJ, countermovement jump; IAT, Illinois agility test.

In order to determine correlation between the input and features (variables) a “Pearson correlation coefficient” was performed to show that how features are related to each other according to data. As shown on the Fig. 5 heat map, by observing the color and value change for each cell along the axis, it can explain how features such as hamstring shortness (group) can correlate with tests like SR and PKET, or how some tests such as SR and PKET are correlated. Analyzing highly correlated features whether in positive or negative relation can help to remove unnecessary features and can show how to perform feature selection and extraction in a way to reduce redundancy of the data to increase the performance of modeling methods.

### Data balancing and augmentation

**Fig. 6:**
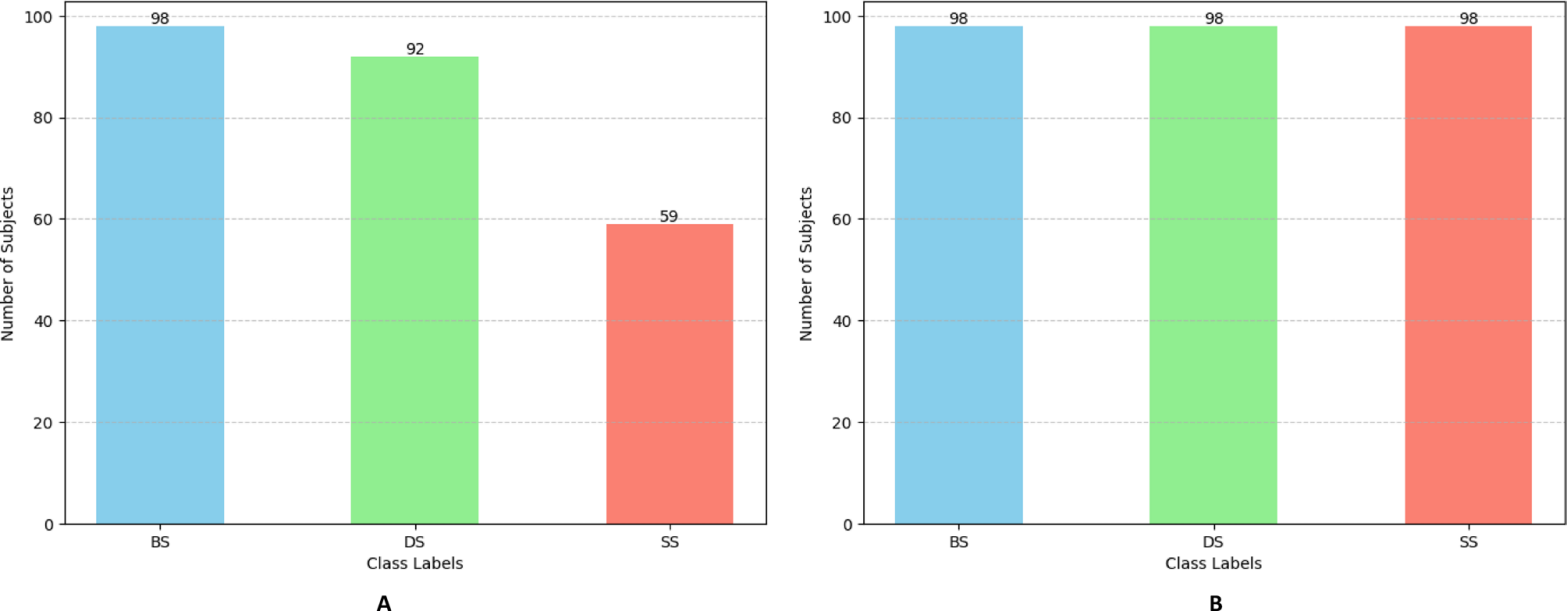
Class distribution of data before and after balancing: **(A)** Original data. **(B)** Balanced data. Abbreviations: SS, static stretching; DS, dynamic stretching; BS, ballistic stretching.

Mostly, an imbalanced data or lack of access to a large dataset can affect model efficiency and decrease model performance. To address this problem, one solution is to balance the data in a way that model can learn on a more balanced environment where there is enough data for each class. In this study we used Synthetic Minority Over-sampling Technique (SMOTE) to balance the data. SMOTE is a data augmentation method that is used to address class imbalance by generating synthetic samples for the minority class. It generates new points by interpolating between each minority example and one of its k-nearest minority neighbors, placing the synthetic instance along the line segment that joins them^48^. As shown in Fig. 6, the minority class has reached the majority class using artificial samples making data more balanced.

### Machine learning techniques

#### Linear discriminant analysis (LDA)

LDA is a supervised dimensionality reduction technique used primarily for classification tasks. LDA aims to find a linear combination of features that best separates two or more classes. It does this task by maximizing the ratio of between-class variance to within-class variance, thereby enhancing class separability. The result is a new set of axes (linear discriminants) that can be used to project the data into a lower-dimensional space while preserving the discriminatory information. It can be used as both classifier and a dimension reduction method^49^.

#### $\:k$-nearest neighbor algorithm (k-NN)

$\:k$-nearest neighbors’ algorithm is a non-parametric supervised learning algorithm first developed by Evelyn Fix and Joseph Hodges, k-NN is consider as a lazy learner and can be used for both classification and regression tasks. when a new data is given to the k-NN model, it calculates the distance between the new data and all the training data points with the given distance measure (mostly Euclidean distance), Then among the k-nearest points to the new data, selects the most frequent class from this k points as the candidate class for the new data^50^.

#### Logistic regression (LR)

logistic regression is a supervised learning method that uses a single perceptron (neuron) for classification and regression. The multiplication of the features of each data in a weight matrix is given to the perceptron as an input, Then, a non-linear activation function will be applied on the summation of the given input. The value of the activation function will be returned as an output, then a loss function calculates the loss of output to update the values of the wight matrix. At last, with a “1 vs all” approach the candidate class for a new data point will be selected^51^.

#### Support vector machine (SVM)

SVM is a supervised ML algorithm which is used for classification tasks. It works by finding the optimal hyperplane that separates data points of different classes in a high-dimensional space. The goal of a support vector machine is to maximize the margin between the closest data points of each class, known as support vectors. This approach not only helps in achieving better classification performance but also enhances the model’s generalization to unseen data. SVM can be extended to handle non-linear classification problems using kernel functions, which transform the input space into a higher-dimensional space where a linear separation is possible^52^.

#### Random forest

A random forest is a supervised ensemble ML algorithm that uses multiple decision trees on randomly sampled subsets of the data and features, then combines their predictions by averaging or voting to improve accuracy and reduce overfitting^53^.

#### Extremely randomized trees (Extra trees)

Extra trees is an ensemble method similar to random forest but uses additional randomness. Instead of searching for an optimal cut and threshold, it selects both the feature and split threshold at random during the construction of the tree. Trees are typically built upon the whole training set, combined with random splits which causes to reduces variance and speeds up training, then combines their predictions by averaging or voting^54^.

### Evaluation of the models

In order to validate the performance of each model for this classification task, a k-fold cross-validation was used with 6-folds which every time each fold selects a random set of data for training and test from the original dataset. data separation rate is around 80% training samples and 20% test samples per fold (Table 2) and the outcome of models was validated using the following validation measurements: Accuracy: $\:accuracy=\:\frac{TP+TN}{TP+TN+FP+FN}$, Recall score: $\:recall=\:\frac{TP}{TP+FN}$, Precision score: $\:precision=\:\frac{TP}{TP+FP}$, F_1_-score: $\:f1=\:\frac{precison\:\times\:\:recall}{precision\:+\:recall}=\:\frac{2\:\times\:\:TP}{2\:\times\:\:TP\:+\:FP\:+\:FN}$ where TP: number of true positives, TN: number of true negatives, FP: number of false positives, FN: number of false negatives.

**Table 2:** Number of distributed data points in train and test sets using k-fold: (A) original data (B) balanced data.

Fold number	Training data	Test data	Total data
**A**			
1	207	42	249
2	207	42	249
3	207	42	249
4	208	41	249
5	208	41	249
6	208	41	249
**B**			
1	245	49	294
2	245	49	294
3	245	49	294
4	245	49	294
5	245	49	294
6	245	49	294

## Supplementary Information

Below is the link to the electronic supplementary material.

Supplementary Material 1

## Data Availability

The data that supports the findings of this study are available in the Zenodo data repository at https://doi.org/10.5281/zenodo.16792671.
