# Supplementary material for: Evaluation of various traditional machine learning techniques for predicting the acute effect of different hamstring muscle stretching methods among male soccer players
Source: Sci Rep. 2025 Dec 4;15:43174. doi: 10.1038/s41598-025-27338-6 (PMC12678761; doi:10.1038/s41598-025-27338-6)
Supplement: Supplementary file 1 — Supplementary Material 1 [file 41598_2025_27338_MOESM1_ESM.docx]

Table S1. Pre- and Post-stretches results for PKET and SR tests, with confidence intervals and effect sizes, in healthy and with hamstring shortness individuals.

| **Groups** | **Protocols** | **Pre PKET**  **M ± SD** | **Post PKET**  **M ± SD** | **With-in group**  **(Cohen’s d; 95% CI)** | **Between protocols**  **(Cohen’s d; 95% CI)** | **Pre SR**  **M ± SD** | **Post SR**  **M ± SD** | **With-in group**  **(Cohen’s d; 95% CI)** | **Between protocols**  **(Cohen’s d; 95% CI)** |
| --- | --- | --- | --- | --- | --- | --- | --- | --- | --- |
| Hamstring shortness | SS | 65.0±2.0 | 71.9±2.7 | 2.91;[6.30]-[7.49] | SS vs. DS: 0.96;[1.84]-[3.15]  SS vs. BS: 0.82;[1.32]-[2.47]  DS vs. BS: 0.27;[0.05]-[1.14] | 19.6±1.0 | 23.1±1.8 | 2.43;[2.80]-[4.19] | SS vs. DS: 0.91;[1.01]-[1.78]  SS vs. BS: 0.26;[0.02]-[0.97]  DS vs. BS: 0.54;[0.48]-[1.31] |
|  | DS | 65.5±1.5 | 69.4±2.5 | 1.81;[3.38]-[4.41] |  | 19.9±0.8 | 21.7±1.2 | 1.71;[1.54]-[2.05] |  |
|  | BS | 65.8±1.9 | 70.0±1.8 | 2.22;[3.73]-[4.66] |  | 19.9±1.9 | 22.6±2.0 | 1.39;[2.21]-[3.19] |  |
| Healthy | SS | 79.9±3.1 | 83.3±1.7 | 1.32;[2.78]-[4.02] | SS vs. DS: 1.08;[1.46]-[2.33]  SS vs. BS: 0.55;[0.61]-[1.58]  DS vs. BS: 0.39;[0.30]-[1.29] | 25.5±0.8 | 28.0±2.0 | 1.64;[2.12]-[2.87] | SS vs. DS: 0.69;0.70-1.49  SS vs. BS: 0.82;[0.83]-[1.56]  DS vs. BS: 0.12;[-0.09]-[0.29] |
|  | DS | 79.6±2.2 | 81.4±1.8 | 0.89;[1.30]-[2.29] |  | 25.6±1.4 | 26.9±1.0 | 1.06;[0.99]-[1.60] |  |
|  | BS | 80.1±3.2 | 82.2±2.2 | 0.76;[1.41]-[2.78] |  | 25.7±1.7 | 26.8±0.5 | 0.87;[0.78]-[1.41] |  |

Abbreviations: SS, static stretching; DS, dynamic stretching; BS, ballistic stretching; PKET, passive knee extension test; SR, sit and reach; M, mean; SD, standard deviation; CI, confidence interval.

Table S2. Pre- and Post-stretches results for hamstring muscle strength tests, with confidence intervals and effect sizes, in healthy and with hamstring shortness individuals.

| **Groups** | **Protocols** | **Pre strength**  **M ± SD** | **Post strength**  **M ± SD** | **With-in group**  **(Cohen’s d; 95% CI)** | **Between protocols**  **(Cohen’s d; 95% CI)** |
| --- | --- | --- | --- | --- | --- |
| Hamstring shortness | SS | 232.4±33.2 | 233.9±21.9 | 0.05;[-5.56]-[8.56] | SS vs. DS: 0.19;[-1.07]-[8.67]  SS vs. BS: 0.06;[-3.24]-[6.64]  DS vs. BS: 0.17;[-1.03]-[6.23] |
|  | DS | 233.2±21.8 | 237.7±16.5 | 0.23;[-0.35]-[9.35] |  |
|  | BS | 232.3±19.4 | 235.1±12.1 | 0.17;[-1.26]-[6.86] |  |
| Healthy | SS | 266.4±18.6 | 266.3±18.9 | 0.01;[-4.55]-[4.75] | SS vs. DS: 0.14;[-1.55]-[5.95]  SS vs. BS: 0.09;[-2.21]-[5.01]  DS vs. BS: 0.08;[-1.46]-[3.06] |
|  | DS | 266.1±6.7 | 268.5±10.0 | 0.28;[0.28]-[4.51] |  |
|  | BS | 267.1±13.8 | 267.7 ±8.2 | 0.05;[-2.21]-[3.41] |  |

Abbreviations: SS, static stretching; DS, dynamic stretching; BS, ballistic stretching; M, mean; SD, standard deviation; CI, confidence interval.

Table S3. Pre- and Post-stretches results for single-leg hop distance and CMJ tests, with confidence intervals and effect sizes, in healthy and with hamstring Shortness individuals.

| **Groups** | **Protocols** | **Pre hop test**  **M±SD** | **Post hop test**  **M±SD** | **With-in group**  **(Cohen’s d; 95% CI)** | **Between protocols**  **(Cohen’s d; 95% CI)** | **Pre CMJ**  **M±SD** | **Post CMJ**  **M±SD** | **With-in group**  **(Cohen’s d; 95% CI)** | **Between protocols**  **(Cohen’s d; 95% CI)** |
| --- | --- | --- | --- | --- | --- | --- | --- | --- | --- |
| Hamstring shortness | SS | 162.6±6.4 | 162.3±3.3 | 0.05;[-0.97]-[1.57] | SS vs. DS: 0.60;[1.40]-[3.39]  SS vs. BS: 0.42;[0.90]-[3.49]  DS vs. BS: 0.03;[-1.20]-[1.60] | 39.7±1.5 | 39.7±1.4 | 0.01;[-0.36]-[0.36] | SS vs. DS: 1.46;[1.83]-[2.56]  SS vs. BS: 0.99;[1.50]-[2.49]  DS vs. BS: 0.09;[-0.30]-[0.70] |
|  | DS | 162.2±2.0 | 164.7±4.5 | 0.71;[1.62]-[3.37] |  | 40.1±1.5 | 41.9±1.5 | 1.19;[1.42]-[2.17] |  |
|  | BS | 162.1±4.6 | 164.5±6.5 | 0.42;[0.98]-[3.81] |  | 40.0±1.4 | 41.7±2.4 | 0.86;[1.20]-[2.19] |  |
| Healthy | SS | 163.1±6.2 | 163.0±2.7 | 0.02;[-1.08]-[1.28] | SS vs. DS: 0.84;[2.12]-[3.88]  SS vs. BS: 0.53;[1.65]-[4.54]  DS vs. BS: 0.01;[-1.45]-[1.65] | 40.6±3.2 | 40.7±2.4 | 0.03;[-0.60]-[0.80] | SS vs. DS: 1.39;[3.12]-[4.47]  SS vs. BS: 1.2;[2.26]-[3.33]  DS vs. BS: 0.39;[0.37]-[1.62] |
|  | DS | 163.5±3.7 | 166.0±4.2 | 0.63;[1.51]-[3.48] |  | 41.7±1.5 | 44.5±3.0 | 1.18;[2.21]-[3.38] |  |
|  | BS | 163.7±4.0 | 166.1±7.8 | 0.38;[0.86]-[3.93] |  | 41.7±2.3 | 43.5±1.9 | 0.85;[1.27]-[2.32] |  |

Abbreviations: SS, static stretching; DS, dynamic stretching; BS, ballistic stretching; CMJ, countermovement jump; M, mean; SD, standard deviation; CI, confidence interval.

Table S4. Pre- and post-stretches results for 30-m sprint and IAT tests, with confidence intervals and effect sizes, in healthy and with hamstring shortness individuals.

| **Groups** | **Protocols** | **Pre**  **sprint**  **M±SD** | **Post**  **sprint**  **M±SD** | **With-in group**  **(Cohen’s d; 95% CI)** | **Between protocols**  **(Cohen’s d; 95% CI)** | **Pre IAT**  **M±SD** | **Post IAT**  **M±SD** | **With-in group**  **(Cohen’s d; 95% CI)** | **Between protocols**  **(Cohen’s d; 95% CI)** |
| --- | --- | --- | --- | --- | --- | --- | --- | --- | --- |
| Hamstring shortness | SS | 7.2±0.1 | 7.3±0.1 | 0.99;[0.07]-[0.12] | SS vs. DS: 2.00;[0.17]-[0.22]  SS vs. BS: 0.99; [0.07]-[0.12]  DS vs. BS: 1.00;[0.07]-[0.12] | 16.7±1.6 | 16.6±1.6 | 0.06;[-0.30]-[0.50] | SS vs. DS: 0.06;[-0.30]-[0.50]  SS vs. BS: 0.06;[-0.29]-[0.49]  DS vs. BS: 0.01;[-0.39]-[0.39] |
|  | DS | 7.2±0.1 | 7.1±0.1 | 1.00;[0.07]-[0.12] |  | 16.7±1.6 | 16.5±1.6 | 0.12;[-0.20]-[0.60] |  |
|  | BS | 7.2±0.1 | 7.2±0.1 | 0.01;[-0.02]-[0.02] |  | 16.7±1.4 | 16.5±1.5 | 0.13;[-0.16]-[0.56] |  |
| Healthy | SS | 6.9±0.1 | 7.0±0.1 | 0.99;[0.07]-[0.12] | SS vs. DS: 2.00;[0.17]-[0.22]  SS vs. BS: 0.99;[0.07]-[0.12]  DS vs. BS: 1.00;[0.07]-[0.12] | 16.0±1.2 | 16.0±1.2 | 0.01;[-0.30]-[0.30] | SS vs. DS: 0.15;[-0.11]-[0.51]  SS vs. BS: 0.27;[0.02]-[0.57]  DS vs. BS: 0.08;[-0.18]-[0.38] |
|  | DS | 6.9±0.1 | 6.8±0.1 | 1.00;[0.07]-[0.12] |  | 15.9±1.3 | 15.8±1.3 | 0.07;[-0.22]-[0.42] |  |
|  | BS | 6.9±0.1 | 6.9±0.1 | 0.01;[-0.02]-[0.02] |  | 15.9±1.1 | 15.7±1.0 | 0.19;[-0.06]-[0.46] |  |

Abbreviations: SS, static stretching; DS, dynamic stretching; BS, ballistic stretching; IAT, Illinois agility test; M, mean; SD, standard deviation; CI, confidence interval.
